# Fracture resistance and bond strength of endodontically treated teeth, restored with three fiber post and core system: an in vitro study

**DOI:** 10.1038/s41598-025-23778-2

**Published:** 2025-11-07

**Authors:** Yasser M. Aly, Samir I. Bakry, Mohammad A. Tuma Halabi

**Affiliations:** 1 Division of Fixed Prosthodontics, Conservative Dentistry Department, Faculty of Dentistry, Alexandria University, Alexandria, Egypt; 2 Department of Fixed Prosthodontics, Faculty of Dentistry, Alexandria University, Alexandria, Egypt; 3 Oral Rehabilitation Sciences Department, Beirut Arab University, Beirut, Lebanon

**Keywords:** Diseases, Health care, Medical research

## Abstract

Fracture resistance of endodontically treated teeth depends on multiple factors including residual dental structure, type of post, diameter, and design of post itself. Aimed to evaluate fracture resistance and push-out bond strength of endodontically treated teeth, restored with three fiber post material and their modes of failure. Twenty-seven newly extracted mandibular second premolars were obtained for this study. Teeth were randomly allocated into three groups based on post type (*n* = 9). Group (RP): Tapered fiber post, group (RGP): Bundle fiber post and group (CP): parallel-sided fiber post. After root canal preparation and obturation, standardized post spaces were prepared and core-build up was done. Fracture resistance and push-out for all samples were evaluated using the universal testing machine under the speed of 0.5 mm/min at 45-degree sloping load which were applied to the restored teeth. The data was collected and statistically analyzed using Kruskal Wallis test. Mode of failure was assessed using a stereomicroscope. There was no statistically significant difference between the three study groups regarding the fracture resistance values (p value 0.552). Kruskal Wallis test revealed that there was no statistically significant difference in push-out values among the three studied groups (p value 0.435). Bundle fiber posts showed similar fracture resistance values in comparison to tapered fiber posts and parallel-sided fiber posts. Regarding fracture resistance and push-out bond strength the highest values were showed in group RP and the lowest values were presented in group RGP.

## Introduction

The rehabilitation of endodontically treated teeth (ETT) continues to be a subject of considerable debate. These teeth are often more vulnerable due to the loss of pulp vitality and structural integrity caused by decay, previous restorative treatments, or endodontic procedures. Consequently, restoring these teeth requires careful selection of materials and techniques to address their inherent fragility and ensure long-term functionality. Moreover, the presence of structural and biochemical changes within tooth structure might hinder the extended duration sustainability of ETT^1,2^.

The prognosis of a tooth depends mostly on the amount of residual dental structure, which is important due to its lack of mechanical properties such as strength, fragility, and susceptibility to fracture. The degree of hard tissue loss and the presence of the residual axial inside walls are pivotal factors in determining the necessity of post placement before final restoration of a tooth. Support was required to ensure the stability of the coronal restoration if adequate tooth structure was still present^1,3,4^.

Prefabricated posts have gained importance due to the variety of systems available, including options that are smooth or serrated, tapered or parallel side, threaded or cemented, or combinations thereof. Metal posts have been a traditional choice for restoring endodontically treated teeth. However, their significantly higher elastic modulus compared to dentin makes them susceptible to fracture. This mismatch in elasticity leads to stress concentration, particularly in the mid-root region, especially under lateral forces, ultimately resulting in structural failure^5,6^.

In the field of dentistry, various types of posts composed of fiber have been created. These include zircon posts, woven polyethylene fiber posts, quartz fiber posts, and glass fiber posts^2^. Fiber reinforced composites have become a viable substitute for conventional materials like glass ceramics, and zirconium in restorative dentistry. Nevertheless, ceramic posts exhibit challenges in being retrieved in retreatment situations due to the need for rotating equipment, which might potentially result in root fracture or perforation^7^. Fiber posts used currently are formed of quartz or glass fibers that are oriented in one direction and inserted in a resin matrix consisting of epoxy resin. The primary advantage of fiber posts is that they have a elastic modulus like dentin, which makes them aesthetically pleasing, especially when paired with all-ceramic crowns. Additionally, fiber posts minimize the danger of root fracture by facilitating smooth bonding devoid of friction against the root canal walls^5,7^.

Rebilda GT and innovative methods like bundle posts offer effective solutions by occupying space within the canals without necessitating the removal of additional dentin. This approach minimizes the thickness of the luting cement required, ensuring a secure fit for the posts while preserving the integrity of the tooth structure. As a result, transfer of tension to root system surfaces is minimized, lowering the possibility of cracks in the vertical roots^5,8^.

Currently, the literature lacks a definitive agreement regarding the most suitable material or type of restoration for effectively restoring ETT^9^.

The bundle post is a composite post that is radiopaque, transparent, and has a similar elastic modulus of dentin. Each post comprises multiple slender individual posts, each approximately 0.3 mm in diameter, organized in varying quantities and encased within a color-coded sleeve for identification^5^.

The adhesive cement used in the cementation procedure possesses a low elastic modulus, which enables it to serve as a shock absorber, effectively reducing the root fractures. This homogeneous biomechanical unit facilitates enhanced homogeneity in the allocation of stress, thereby preserving weakened tooth structure more effectively^10^.

There is limited agreement in the literature that one material is advantageous over another concerning its mechanical behavior and mode of failure. Thus, this study aimed to evaluate fracture resistance, push-out, and mode of failure of bundle fiber post, tapered fiber post and parallel-sided fiber post. The null hypothesis is that there will be no difference in fracture resistance, push-out and mode of failure on bundle fiber post, tapered fiber post and parallel-sided fiber post.

## Materials and methods

### Ethics, consent to participate, and consent to publish

The study was initiated after getting ethical approval from the Scientific Research Committee at the Faculty of Dentistry, Alexandria University (IORG0008839), Ethics Committee No (0764-09/2023). The study was carried out following the Declaration of Helsinki, along with all participants giving written informed consent before participating in the study.

### Consent to participate

Prior written informed consent was obtained for the collection of teeth used in this study.

A sample size of 27 was calculated based on a previous study^11^, using statistical software (G*Power v3.1.9.2; Heinrich-Heine-Universität Düsseldorf)^12^, where α value was set at 0.05, and the power was set at 80%. The minimum sample size was determined to be 9 per group.

Three groups were examined in this in-vitro, parallel-controlled study to measure fracture resistance, bond strength and failure mode using statistical software (IBM SPSS, version 23; Armonk) (3 groups; *n* = 9 per group).

This study was an in vitro analysis that assessed fracture resistance, failure mechanism, and push-out bond strength across three tested groups. The patient consented to the utilization of their teeth for this study. It was held at the laboratory of Conservative Dentistry Department at the Faculty of Dentistry, Alexandria University, Egypt. For this study, 27 newly harvested mandibular second premolars were gathered and preserved in thymol solution at a 0.1% concentration until it becomes operational^5,13^. All extracted teeth were selected with following criteria, intact root, fully mature single roots and examined under optical microscope (Olympus, Tokyo, Japan) to exclude any tooth with multiple root canals, root cracks, open apices, fractures, resorptive defects, or previous root canal treatment^14,15^. Digital periapical radiographic imaging was performed for each sample to confirm the alignment and straightness of the root canal pathways. The dimensions of each specimen were assessed using a precise measuring insize digital caliper (Insize Co., Ltd., Binjiang District, Zhejiang, China) to verify a root length of 14 ± 1 mm from the cemento-enamel junction to the apex, measurements were carefully taken^14,15^.

The root lengths of the chosen teeth were assessed from the cement-enamel junction to the apex using digital caliper to determine their measurements accurately (Insize Co., Ltd., Binjiang District, Zhejiang, China); they were 14 mm long (± 1 mm)^16^. A periodontal curette (Nordent Manufacturing Inc., T: 800.966.7336 US & Canada) was used to eliminate soft tissue. Prior to root canal preparation, a double-sided diamond disc (komet Dental. Gebr. Brasseler GmbH Germany) was used attached on straight handpiece (NSK EX-203 C, Japan). The remaining root average length was adjusted to the cement-enamel junction (CEJ) level under continuous coolant. The teeth were positioned in acrostone acrylic resin blocks, each having a diameter of 15 mm and a height of 18 mm (Acrostone Dental Manufacturer, 215 El Hegaz St., Heliopolis, Cairo, Egypt) in the teeth were placed in metallic molds with their long vertical axis aligned. Above each block, 2 mm of the coronal structure was visible. Acrylic excess material was eliminated prior to full settling^3,17,18^.

Regarding endodontic procedure the Endo-Z and Rose-head bur (Komet Dental. Gebr. Brasseler Germany) were affixed to a high-speed turbine to prepare access cavity. The process of determining the working length of the root canal involved inserting a number 10k-file instrument until it reached the apex. Each specimen was ensured for its length using periapical radiograph imaging^3,11^.

Pro-taper next (Maillefer-Dentsply, Switzerland) was used for cleaning and shaping process of root canal up to the required working depth by using Endomotor (Wismy, Guangdong, China) at 250 rpm with 2 N/cm torque^3^.

Regarding irrigation, use a 30-gauge needle with side openings for irrigation (#1707 Yinqiao Building, 58Jinxin Rd, Pudong, Shanghai 201206, China) with a 2.5% sodium hypochlorite solution for canal irrigation^3^. Following drying process with F2 paper points until full dryness of the channels was obtained^14^. Canals were filled with single cone F2 gutta-percha through lateral condensation at working length with minimal resistance and endodontic ADSEAL (Meta Biomed Co., Korea). The access cavities were temporarily filled with Tetric N-ceram nanohybrid composite and kept in an incubator at 37 °C with 100% humidity for a duration of one week^3,5,11^.

The total number of specimens was randomly^11^, selected and divided into three groups according to post material. **Group RP**: 9 teeth restored with tapered fiber post (Rebilda) with 1.2 mm diameter and 4 mm composite resin core. **Group RGP**: 9 teeth restored with bundle fiber post (Rebilda GT) with 1.2 mm diameter and 4 mm composite resin core. **Group CP**: 9 teeth restored with parallel-sided fiber post (Glassix) with 1.2 mm diameter and 4 mm composite resin core as mentioned in Table 1 showed brand, manufacturer, and composition materials used in this study.

**Table 1:** Brand, manufacturer, and composition materials used in this study.

Product	Composition	Manufacturer	Lot. number
Resin cement Bifix SE	Barium aluminium glass, GlyDMA, UDMA, Methacylate phosphoric acid ester, BisGMA, Fumed silica, HPMA, BisEMA, Initiator, Stabilizers, Pigments	VOCO, Cuxhaver, Germany.	2,334,490
Solobond M bonding agent	Aceton, BisGMA, HEMA, Methacylate phosphoric acid ester, Methacylate functionalized polyalkenoic acid, HPMA, Stabilizers		2,247,140
Rebilda Post	urethane-dimethacrylate (UDMA) 70 wt%; glass fibre 20 wt% and glass filling 10 wt%		2,232,569
Rebilda GT Post	70% glass fiber, 10% inorganic filler, 20% DMA matrix		2,309,114
Self-cure acrylic resin	Polymethyl methacrylate (PMMA)	Acrostone Dental Manufacturer, 215 El Hegaz St., Heliopolis, Cairo, Egypt.	
Silane coupling agent: maquira	Silane and ethanol	Maquira Industry of Dental Products Ltd., Brazil.	988,722
Glassix post	Glass fiber, epoxy resin matrix	Glassix, Nordin, Switzerland	22,179

After randomly assigning the specimens into groups, the access cavities were accessed again by completely eliminating the temporary filling material. Post space preparation was subsequently carried out by removing the gutta-percha from the upper 2/3 of the root canal, around 10 mm in depth, while preserving around 4 mm of gutta-percha at the apical region to establish an effective seal, by using Peeso reamer (Mani, Inc.,Japan) drills, which were attached to a low-speed turbine w&h (Ignaz-Glaser-Str.53,5111 Bürmoos Austria), the length was meticulously adjusted using an endodontic ruler. These instruments were then utilized in the prescribed sequence: #1, #2, and #3, in order to achieve uniformity in post space preparation^3,5,16^.

### For the tapered post space Preparation (Group RP)

The Rebilda fiber post system (Voco, Cuxhaven, Germany) used the #2 shaping drill, with 1.2 mm diameter, to prepare the post space. The drill length was adjusted to 10 mm to standardize the preparation process^3,5,16^.

### For the parallel-sided and bundle post space Preparation (Group CP & RGP)

The #2 shaping drill from the Glassix fiber post system (Glassix, Nordin, Switzerland) was used, with 1.2 mm diameter, to prepare the post space, with the drill’s length adjusted to 10 mm to standardize the procedure^3,5,16^. Following the manufacturer’s guidelines, the Rebilda GT glass fiber post system (Voco, Cuxhaven, Germany) was used, which is compatible with all drill systems^5^.

For cementation process, the posts were treated with alcohol for cleaning and subsequently dried using compressed air. Silane coupling agent (Maquira Industry of Dental Products Ltd., Brazil) was treated to the post for 60 s and subsequently dried using compressed air. The post spaces were irrigated with normal saline, 70% alcohol solution, and 2.5% sodium hypochlorite. Subsequently, they were dried completely by endodontic absorbent paper points (Meta Biomed Co. Ltd, Korea) and a self-adhesive bonding agent Solobond M (Voco, Cuxhaven, Germany) was applied into post space to ensure proper adhesion. Bifix SE, a dual-curing luting cement (Voco, Cuxhaven, Germany) following the instructions provided by the company, an auto-mixing tip was utilized to ensure accurate cement delivery into the post spaces and even distribution of the cement across the post surfaces before insertion into post space. Then post inserted into post space under static load device using a load of 2 kg till excess cement was extruded. The cement mixture underwent light polymerization 20 s and 4 min for chemical-polymerization as a final setting duration according to manufacture instructions. After the tapered, parallel-sided, or bundle posts were cemented, the cores were constructed through specially designed crown former of 4 mm inciso-cervically on thermoplastisized sheet to ensure standardization using a dual-cure adhesive composite resin Charmcore (Dentkist, South Korea) core foundation system and then finished using diamond rotary tools^5,16,19^.

**Fig. 1:**
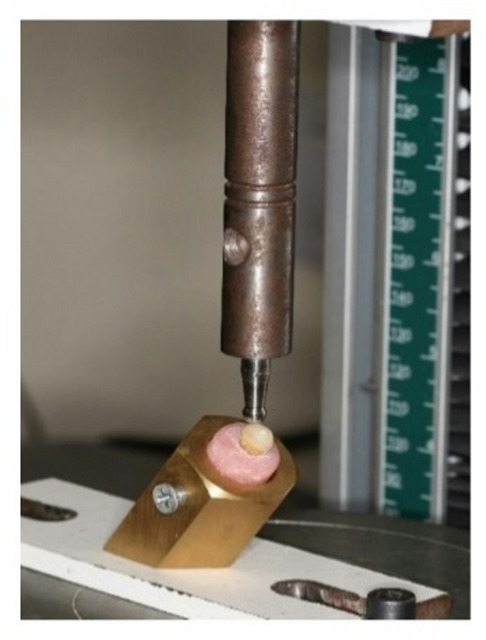
Specimen under universal testing machine for fracture resistance test.

Each specimen was attached to a universal test device (5st, Tinius Olsen, England) at a speed of half millimeters per minute. The tooth within the acrylic block was attached to a special jig which is designed at 45 degrees and was secured to the base of a universal testing machine, where a vertical load was applied to the outer cusp inclines. The extremely force necessary to induce failure was recorded in Newtons (N) (Fig. 1)^20^.

The failure modes were observed under a stereomicroscope (SZ1145TR Olympus; Japan 1990) and categorized into favorable fracture and unfavorable fracture, or any form of interface debonding^21^.

**Fig. 2:**
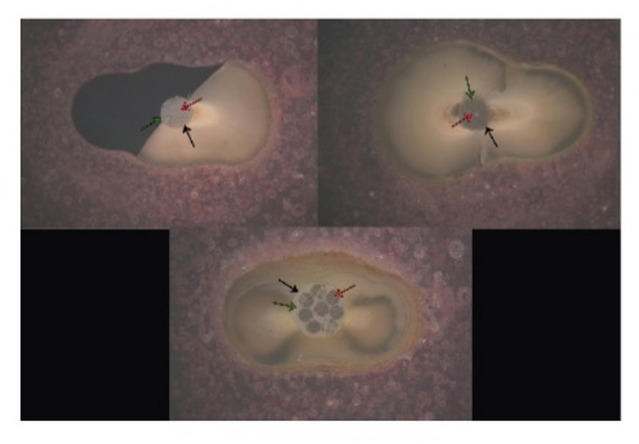
Stereomicroscope images illustrate middle section before push-out test Green arrow: resin cement, Red arrow: fiber post, Black arrow: dentin.

For push-out test, specimens were inserted longitudinally in self-curing acrylic blocks and subsequently sectioned into 1 mm thick slices using a precision-cutting device equipped with water-cooling functionality (IsoMet 4000, Buehler USA) at speed of 2500 rpm. From each specimen, six sections were obtained: two apical, two middle and two coronals (Fig. 2).

**Fig. 3:**
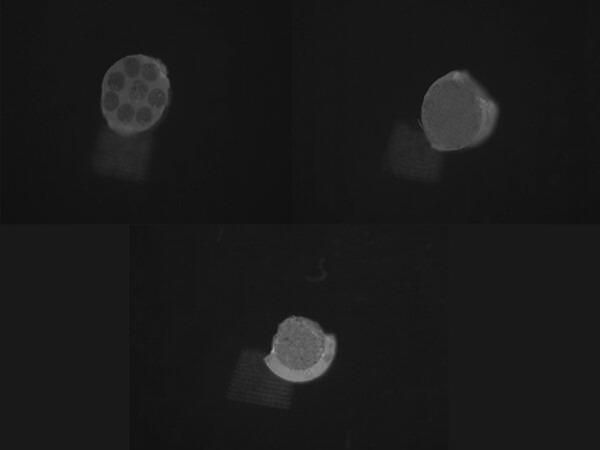
Stereomicroscope image of mixed failure (mixture of adhesive in dentin and cohesive in resin cement).

Each segment was identified using a permanent marker for clear differentiation to differentiate between the apical and coronal segments. The bond strength of each specimen was evaluated using a universal testing apparatus (5st, tinius olsen, England), employing a cylindrical plunger measuring 1 mm in diameter was used, operating at a crosshead speed of 0.05 mm/min. The highest load at failure was recorded in Newtons (N), and a determination was made by utilizing the equation A = 2r × π × h^22^ to ascertain the bonding surface area (A). Where r represents the radius of the post, h represents the thickness of each segment of the post, and π represents the constant of 3.14. Then The bond strength (δ), expressed in megapascals (MPa), was subsequently determined using the equation $\:\delta\:=\frac{F}{A}$​^21–23^. A stereomicroscope was utilized in order to investigate the various modes of failure. for detailed observation (SZ1145TR Olympus; Japan 1990) under a magnification of 50X (Fig. 3); and categorized into distinct classifications including cohesive, adhesive and mixed^24^.

### Statistical analysis

Data were analyzed using IBM SPSS version 23 for Windows, Armonk, NY, USA. Normality of fracture resistance and push out test were assessed using Shapiro Wilk test and Q-Q plots. Non-normal distribution was confirmed for all variables thus median, minimum and maximum were used for data presentation in addition to mean and standard deviation (SD). Mode of failure was presented using frequency and percentage. Comparison between groups regarding fracture resistance and push out test was done using Kruskal Wallis test while Chi Square test was used to assess differences in mode of failure between groups. All tests were two tailed and the significance level was set at *p* value < 0.05.

## Results

Table 2 showed the comparison of the fracture resistance between the three study groups, Group RP had a median score of 490.61 N, with values ranging from 204.97 N to 999.54 N, Group CP had a median score of 424.52 N, with values ranging from 102.33 N to 570.86 N and Group RGP had a median score of 394.71 N, with values ranging from 168.21 N to 827.09 N. No statistically significant difference between the three study groups regarding the fracture resistance values (p value 0.552).

**Table 2:** Comparison of fracture resistance among the study groups.

	Group I (RP) (*n* = 9)	Group II (RGP) (*n* = 9)	Group III (CP) (*n* = 9)
Mean ± SD	535.21 ± 253.10	447.70 ± 188.67	396.42 ± 154.47
Median	490.61	394.71	424.52
Min – Max	204.97–999.54	168.21–827.09	102.33–570.86
H test (*p* value)	1.187 (0.552)		

H test: Kruskal Wallis Test.

**Fig. 4:**
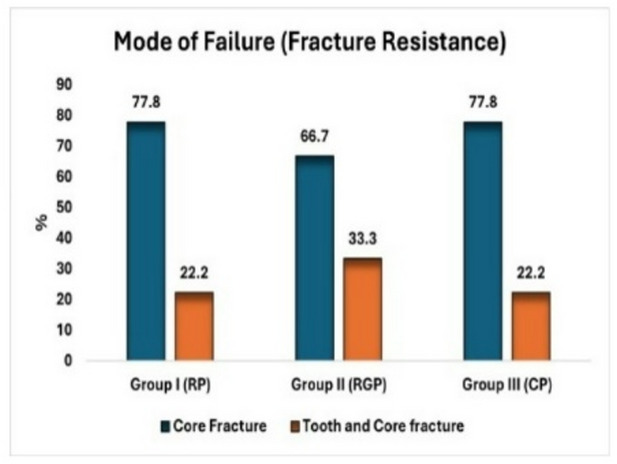
Showing mode of failure (fracture resistance).

**Fig. 5:**
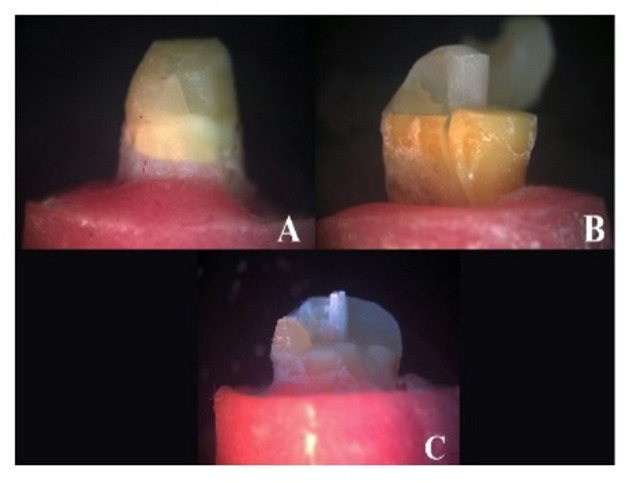
A Showing favorable type of failure (core fracture). B&C Showing catastrophic type of failure (tooth and core fracture).

In the RP and CP groups, 77.8% of the specimens displayed a favorable fracture (core fracture), while 22.2% displayed an unfavorable fracture (tooth and core fracture), with no post-fracture. In the RGP group, 66.7% of the specimens displayed a favorable fracture (core fracture), while 33.3% displayed an unfavorable fracture (tooth and core fracture), with no post-fracture observed. There was no statistically significant difference between the three study groups regarding the fracture mode of failure (p value 0.852) as illustrated in (Figs. 4 and 5).

Table 3 presented a comparison of push-out bond strength among the three experimental groups. The analysis revealed no statistically significant differences in push-out values across the groups (*p* = 0.435).

**Table 3:** Comparison of push-out among the study groups.

	Group I (RP) (*n* = 9)	Group II (RGP) (*n* = 9)	Group III (CP) (*n* = 9)
Mean ± SD	7.32 ± 2.88	5.96 ± 1.15	5.94 ± 3.24
Median	8.20	6.33	5.29
Min – Max	3.37–9.50	4.30–7.06	1.55–10.32
H test (*p* value)	6.343 (0.435)		

H test: Kruskal Wallis Test.

**Fig. 6:**
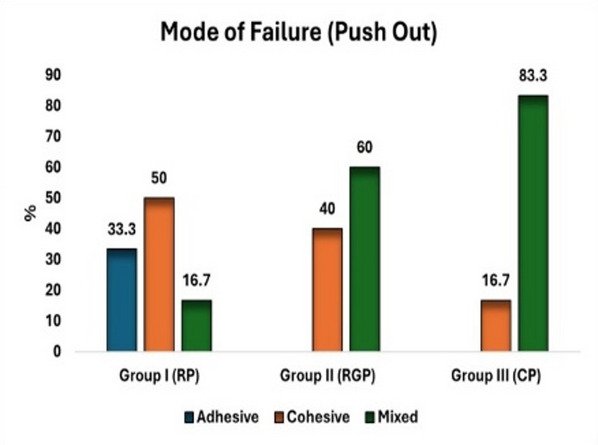
Showing mode of failure (push-out).

The findings demonstrated no statistically significant differences among the three experimental groups in terms of the failure mode related to push-out bond strength (*p* = 0.124), as illustrated in (Fig. 6).

## Discussion

Restoring teeth after endodontic therapy is a highly important aspect of dental practice. Using a post and core is a frequently employed method for repairing damaged teeth. To achieve successful repair, it is necessary to provide particular concern to the structural, functional, and aesthetic aspects. This involves offering enough support for a core, which acts as the essential foundation for the final restoration^1^.

This study exclusively employed single rooted premolars due to their established susceptibility to fracture under occlusal loading subsequent to endodontic treatment. Additionally, the application of loading forces at a 45-degree angle introduces extra-axial forces onto the restored tooth, generating unfavorable stress during functional tasks. According to the findings of Wandscher et al.^10,25^, oblique loading represents a critical scenario for evaluating the fracture resistance of endodontically treated teeth (ETT), as it imposes significant mechanical stress that closely mimics clinical challenges^10,25^.

In this study, the canal preparation was done for all specimens with 10 mm length leaving 4 mm of gutta-percha at the apex ensures that a satisfactory apical barrier^26^. Which is in accordance with Amarnath GS et al.^6^, who stated that extending the post length more than two-thirds of the root results in elevated stresses apically, potentially jeopardizing the integrity of the root’s apical seal. Thus, preserving 3 to 6 mm of gutta-percha at the apical region is essential to maintain the integrity and functionality of the apical barrier. Similarly, the findings of Chuang et al.^27^, revealed that increasing the length of the post could adversely affect the structural integrity of the root by reducing its strength.

The present study assessed resistance to fracture of endodontically treated mandibular second premolars, restored with three different posts, specifically in relation to post spaces with a diameter of 1.2 mm., research findings suggest that the diameter of the post material plays a more significant role than its length in determining fracture resistance. Increasing the diameter of the post can improve fracture resistance; however, it may also compromise the remaining tooth structure’s ability to resist stress due to the excessive removal of healthy dentin. This trade-off underscores the need for a balance between post diameter and the preservation of tooth integrity^3^.

In post cementation procedures, dual-cure resin cements are recognized for their reliability in achieving uniform polymerization throughout the post canal. Despite their ability to polymerize chemically in the absence of light, their physico-mechanical properties are significantly improved with adjunctive light activation. Consequently, the combined use of chemical and photo-curing is routinely advocated to optimize the performance of these materials^28^.

Additionally, Merve Özarslan et al.^3^, showed that the diameter of the post plays a more significant role in determining fracture strength than the length of the post. This suggests that increasing the post diameter has a greater impact on the structural integrity of the restoration compared to merely adjusting the post length. Increasing the post diameter can enhance its resistance, but it may also necessitate the removal of excessive intact tooth tissue, thereby weakening the overall structure. Conversely, if the post diameter is too small, the cement layer thickens, this leads to unfavorable stress distribution at the interface between the post, cement, and dentin, which may result in a loss of retention of the post. Such stress misalignment can weaken the bond between the post and surrounding dentin, making it more susceptible to failure.

The bond strength between posts and dentin is affected by various factors, including the material composition of the post, its shape, and the type of luting agent used. Rebilda GT represents an innovative class of glass fiber dental posts characterized by a bundled configuration of glass fiber-reinforced composite posts. Rebilda GT effectively occupies the canal space and conforms intimately to the canal walls. Consequently, it emerges as a promising solution for challenging cases involving sharply curved, oval, or irregularly shaped root canals, characterized by significant conicity^5^.

The luting cement employed in this study remained consistent throughout all experiments for the three groups to eliminate any significant variations. Naumann et al.^4^, showed that Bifix SE exhibits superior retention levels even after one year, retaining excellent mechanical properties despite prolonged exposure to stress. Naumann et al.^4^, reported that bonded fiber and titanium posts employed for the restoration of endodontically treated teeth showed a mean survival period of up to 100 months, regardless of the material and rigidity of the posts^24^.

In this study, constructing a crown would have potentially skewed the results related to fracture resistance, as it would redirect the compressive forces into tooth directly rather than post. Since the primary focus was on evaluating the fracture resistance of the post restoration, it was crucial to apply the load directly to the core. This approach ensures that the majority of the forces are transmitted directly to the core then post, allowing for a more accurate assessment of the post’s structural performance under load^29^.

Based on the findings of this study, the null hypothesis, which proposed no significant difference in fracture resistance among the groups examined, was upheld.

The fracture strength of a post restoration is determined by several factors, including post design, the diameter and length of the post, as well as the type of adhesive cement used.

These factors facilitate the distribution of occlusal forces from the post to the remaining radicular tooth structure, reducing stress concentrations and enhancing the stability of the restoration^3^. Therefore, this study found no statistically significant difference among the three groups.

Although the mean fracture resistance reached its peak value in group RP (535.21 ± 253.10 N), then CP group (447.70 ± 188.67 N) and the group RGP exhibited the lowest recorded mean fracture resistance among the studied groups (396.42 ± 154.47 N). This might be due to the force was directed on the core and the coronal third of the root, no force was transmitted into post itself^30^, or it might be due to the minimal elastic modulus aids in the distribution and absorption of forces along the lengths of both bundle and fiber posts. Additionally, their ability to bond effectively with tooth dentin contributes to the creation of a monoblock effect^19^.

The fracture resistance of teeth rehabilitated with glass fiber post systems demonstrated considerable variability, which may be attributed to pre-existing structural defects and the inherently low fracture toughness characteristic of glass fiber posts^31^.

These various glass fiber post types share a similar level of rigidity due to the alignment of fibers in a longitudinal direction within the epoxy resin. This arrangement helps dissipate stress by allowing the fibers to slightly adjust their orientation when subjected to load^31^.

The results of this study were comparable to the results presented by Alhanoof Aldegheishem et al.^32^, Esra Kul et al.^19^, and Ranjkesh B et al.^33^, who also investigated the fracture resistance of parallel-sided and bundle posts posts. The experiment revealed no statistically significant differences in fracture resistance among the groups tested. Both bundle posts and fiber posts exhibited equal fracture resistance values.

This finding contrasts with Alkhalidi EF, who reported that bundle fiber posts demonstrated significantly higher fracture resistance than single glass fiber posts. The superior performance of bundle fiber posts was attributed to their enhanced bonding with the resin matrix, enabling more effective stress distribution compared to single posts^5^.

Push-out testing was conducted on all specimens in this study to determine the maximum force necessary for debonding. The technique was chosen because it effectively reduces the negative effects of tensile and shear stresses, which can cause uneven forces on the tooth surface due to large bonded area of the posts^34^.

As a result, push-out tests demonstrated reduced bonded surface areas, lack of early failures, and verified that the applied force was aligned parallel to the interface^34^. Thus, in this study, mean comparison of push-out among the study groups, the higher mean value was in group RP 7.32 ± 2.88 Mpa followed by group RGP was 5.96 ± 1.15 Mpa then CP group 5.94 ± 3.24 Mpa respectively. No statistically significant differences were observed among the three study groups in the push-out bond strength test. (p value 0.435).

As in previous study by Bitter et al.^28^, assessed that posts composed of bundled fibers exhibit a greater density of internal voids compared to their solid counterparts.

A prior investigation by Hani M. Mouafaq et al.^35^, assessed the push-out bond strength of various fiber post systems. The study found no statistically significant differences between the groups, consistent with the results of the present research. The authors attributed this finding to the unique design of the Rebilda GT system, which incorporates a bundle configuration. This structure enhances the surface area available for luting and facilitates improved stress distribution^35^.

Utar M. et al.^36^, investigated the push-out bond strength of prefabricated glass fiber posts compared to bundled glass fiber posts and reported no significant differences in bond strength between the two. The authors suggested that the design of the post plays a critical role in determining bond strength and retention within the root canal. They noted that the number of fibers in the composite fiber bundle could influence the thickness of the resin cement. Unlike solid posts, which are fully encased in cement, the application of cement around bundled fibers is interrupted by the fibers embedded within the resin matrix, potentially impacting the bond interface^36^.

Conversely, Alves et al.^37^, reported that the bundle post system alone did not demonstrate adequate bond strength for weakened roots. However, when combined with a single post, the system yielded more favorable results, suggesting that the hybrid approach may enhance performance in such scenarios.

In regarding mode of failure, this study showed the highest value of favorable mode of fracture in groups RP and CP was 77.8%. This observation differs from the findings of Alhanoof Aldegheishem et al.^32^, who reported a higher proportion of teeth with favorable fractures in the bundle post group (77.7%) compared to the control group (66.6%) and the fiberglass post group (33.3%)^32^.

In contrast, a study by Kim et al.^29^, reported that 55.6% of specimens exhibited unfavorable fractures. This outcome may be attributed to the use of crown restorations with a 2 mm ferrule, where the majority of compressive forces are transferred directly to the crown margin and tooth structure rather than the post and core material.

The results of this study suggest that both post systems contribute to enhanced fracture resistance in endodontically treated teeth (ETT). However, the study did not fully replicate clinical conditions, such as the thermal variations and masticatory forces typical of the oral cavity. Additionally, the investigation did not assess the applicability of these systems across different types of teeth. Future research should focus on more comprehensive clinical simulations, including dynamic loading and long-term clinical trials, to provide a deeper understanding of the bundle post system’s performance in restoring ETT.

## Conclusion

From the parameters of this study, the subsequent findings were derived:

The findings revealed no statistically significant difference in fracture resistance or push-out bond strength between endodontically treated teeth restored using either a bundle post or a single post. Among all tested groups, favorable types of failure were predominantly observed.

## Data Availability

The data related to this study will be made available upon request from the corresponding author.

## Abbreviations

mm: Millimeter
IBM SPSS: Software for advanced statistical analysis
N: Newton
MPa: Megapascal
mm/min: Millimeters per minute
°C: Celsius or centigrade
RGP: Bundle fiber post
RP: Tapered fiber post
CP: Parallel-sided fiber post
ETT: Endodontically treated teeth
%: Percent
CEJ: Cement-enamel junction
N/cm: Newtons per centimeter
rpm: Revolutions per minute
Kg: Kilogram
SD: Standard deviation
